# The PULSE Project: A Case of Use of Big Data Uses Toward a Cohomprensive Health Vision of City Well Being

**DOI:** 10.1007/978-3-030-51517-1_39

**Published:** 2020-05-31

**Authors:** Domenico Vito, Manuel Ottaviano, Riccardo Bellazzi, Cristiana Larizza, Vittorio Casella, Daniele Pala, Marica Franzini

**Affiliations:** 8 Digital Research Centre of Sfax, Sfax, Tunisia; 9 Institut Mines-Télécom, CNRS, Paris, France; 10 Université de Sherbrooke, Sherbrooke, QC Canada; 11 Digital Research Centre of Sfax, Sfax, Tunisia; 12 University of Sfax, Sfax, Tunisia; 13 Centre of Health Technologies, Università degli studi Pavia, Via Adolfo Ferrata 5, 27100 Pavia, Italy; 14 E.T.S.I. de Telecomunicació, Universidad Politécnica de Madrid, Madrid Av. Complutense, 30, 28040 Madrid, Spain; 15 Department of Civil Engineering and Architecture, Università degli studi Pavia, Via Adolfo Ferrata 5, 27100 Pavia, PV Italy

**Keywords:** Air pollution, Health, Data platform, Participation

## Abstract

Despite the silent effects sometimes hidden to the major audience, air pollution is becoming one of the most impactful threat to global health. Cities are the places where deaths due to air pollution are concentrated most.

In order to correctly address intervention and prevention thus is essential to assest the risk and the impacts of air pollution spatially and temporally inside the urban spaces. PULSE aims to design and build a large-scale data management system enabling real time analytics of health, behaviour and environmental data on air quality. The objective is to reduce the environmental and behavioral risk of chronic disease incidence to allow timely and evidence-driven management of epidemiological episodes linked in particular to two pathologies; asthma and type 2 diabetes in adult populations. developing a policy-making across the domains of health, environment, transport, planning in the PULSE test bed cities.

## Introduction

Air pollution has become silently and hiddendly one of the most impactful menace to global health.

The European Environmental agency [1] estimates that premature deaths attributable to exposure to air pollution of fine matter particles reach are about 412 000 in over 41 EU countries. The exposure to NO2 and O3 concentrations on the same countries in 2016 has been around 71000 and 15000 respectively.

The health threat of air pollution remain located mostly in cities. But the effects does not only limitate on wellbeing, but are also econonomical. The most vulnerable to the risks are lower income socio-economic groups that nowadays are also the most exposed to environmental hazards.

Air pollution indeed does not represent only a sanitary issue: it’s burden reflects also in increasing medical costs.

Air pollution thus, is a problem can be only addressed with a strategic vision can only be addressed with long term targeted policies, majorly in urban environments.

In the year 2015 ITU and the United Nations Economic Commission for Europe (UNECE) gave the definition of smart and sustainable city as “an innovative city that uses information and communication technologies (ICTs) and other means to improve quality of life, efficiency of urban operation and services, and competitiveness, while ensuring that it meets the needs of present and future generations with respect to economic, social, environmental as well as cultural aspects”. This definition led also in 2016, in the United for Smart Sustainable Cities initiative (U4SSC). This open global platform responded to United Nations Sustainable Development Goal 11: “Make cities and human settlements inclusive, safe, resilient and sustainable.”, offering an enabling environment to spread knowledge and innovation globally [2]. Also the health sector has been contaminated by this vision: the increase of social networking, cloud-based platforms, and smartphone apps that support data collection has enhance opportunities to collect data outside of the traditional clinical environment. Such informative explosion allowed patients to collect and share data among each other, their families and clinicians.

Patient-generated health data (PGHD) is defined as health-related data generated and recorded by or from patients outside of the clinical areas. This data could be an important resource available for patient, clinicians and decision makers to be used by to address a current or emerging health issue, and most of it is globally wide, also if they are integrated by information coming from diffuse sensory/IoT devices and Manually input voluntary data reported by the patients, caregivers, or generic citizen participation bring to shared decision-making. The definitions above helps to understand the context of PULSE project. PULSE aims to design and build a large-scale data management system enabling real time analytics of flows of personal data.

The objective is to reduce the environmental and behavioral risk of chronic disease incidence to allow timely and evidence-driven management of epidemiological episodes linked in particular to two pathologies; asthma and type 2 diabetes in adult populations. Developing a policy-making across the domains of health, environment, transport, planning in the PULSE test bed cities.

The project is currently active in eight pilot cities, Barcelona, Birmingham, New York, Paris, Singapore, Pavia, Keelung and Taiwan, following a participatory approach where citizen provide data through personal devices and the PulsAIR app, that are integrated with information from heterogeneous sources: open city data, health systems, urban sensors and satellites. PULSE foster long-term sustainability goal of establishing an integrated data ecosystem based on continuous large-scale collection of all stated heterogeneous data available within the smart city environment.

## The PULSE Project

PULSE project is goaled on build a set of extensible models and technologies to predict, mitigate and manage health problems in cities and promote population health.

Currently PULSE is working in eight global cities. It harvest a multivariate data platform feed by open city data, data from health systems, urban and remote sensors and personal devices to minimize environmental and behavioral risk of chronic disease incidence and prevalence and enable evidence-driven and timely management of public health events and processes. The clinical is on asthma and Type 2 Diabetes in adult populations: the project has been pioneer in the development of dynamic spatio-temporal health impact assessments through exposure-risk simulation model with the support of WebGis for geolocated population-based data.

PULSE gives finally a more wide vision of wellbeing were it is intended also in the relationship with environmental conditions.

### Data Collection Principles

Acquisition, systematization and correlation of large volumes of heterogeneous health, social, personal and environmental data is among the core and primary activities in the PULSE project.

The overall goal of the deployments involves deriving additional values from the acquired data, through: developing more comprehensive benchmarking and understanding of the impact of social and environmental factors on health and wellbeing in urban communities, thereby broadening the scope of public health.

On this sake PULSE has developed tools for end-users (primarily citizens and patients, public health institutions and city services) that leverage open, crowd-sourced and remote sensing data, through integration, enrichment and improved accuracy/reliability of risk models, to guide actions and deliver interventions aiming to mitigate asthma and T2D risk and improve healthy habits and quality of life.

**Fig. 1.**
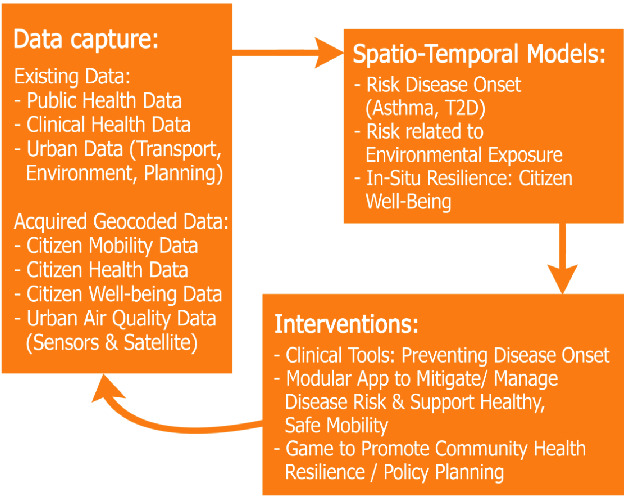
PULSE data flow conceptual structure. The figure shows the collected data clustered on the basis of macro pourpouses

Figure 1 shows the conceptual schema of the relationships among dataflows.

### Clinical Focus

PULSE project focuses on the link between air pollution and the respiratory disease of Asthma, and between physical inactivity and the metabolic disease of Type 2 Diabetes. The risk assessment for this two pathologies comprises the evaluation respectively of:

- *for type 2 diabetes:* behavioural risks associated (i.e. reduced exercise/physical activity at home or in public places). This is associated with higher risk of T2D onset in a dose-response relationship. The assessment use unobtrusive sensing/data collection and volunteered data to collect baseline measures of health and wellbeing, and tracking and model mobility at home and across the city (including time, frequency and route of mode of transit and/or movement).
- *for asthma:* Environmental/exposure risks (i.e. exposure to air pollution, especially with regard to near roadway air pollution). Poor air quality is associated with higher risk of Asthma onset and exacerbation.

Risks of diseases onset are evaluated thorough risk assessment models, that in PULSE are biometric simulation models that predict the risk of the onset of the ashtma and diabetes in relationship to air quality.

The models has been developed by chosen ones from a literature review of the prediction models of type 2 diabetes (T2D) onset and asthma adult-onse. Some of them were selected to be implemented and recalibrated on the datasets available on PULSE repository and adding new variables [12].

### Data Architecture

PULSE architecture is composed by 5 main structures [15]: PULSEAir, App Server, AIR Quality distributed sensor system, GisDB, WebGIS and Personal DB.

- *PULSE App:* is the personal App provided to the participants in charge of collecting sensors data and interacting with the users to propose interventions and gamification. PulsAIR is available both for iOS and Android and can be connected to FitBit, Garmin and Asus health tracker devices.
- *AIR Quality distributed sensor system:* the PULSE air quality sensor’s system is composed of multiple type of sensors and sensor’s datasets: it combines mobile sensors and mobile network of sensors in order monitor the variable trends in emission within urban areas with an high resolution and to appropriately address the temporal and spatial scales where usually pollutants are spread. Two types of sensors has been used across pilots that are the AQ10x of DunavNet (20+, deployed in all pilots) and PurpleAir PA-II sensor.
- *App Server*: This structure internally connects PULSE components.
- *Personal DB*: This repository contains personal detail, connectivity, activity logs, pilot sites structured data, etc.…
- *GISDB*: This repository is in charge of collecting non-personal sources of data: satellite, open repositories and fixed sensors.
- *WebGIS*: This data engine is in charge of aggregating and exploiting all the collected data to build front-end visualizations through maps.

## Well Being Model and Urban Wellbeing

**Fig. 2.**
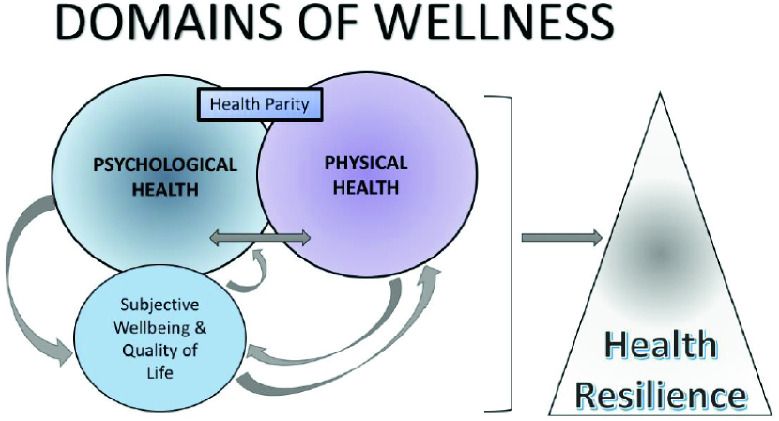
Model of the domains of wellness

The WHO definition of health includes reference to wellbeing: health is “a state of complete physical, mental and social wellbeing and not merely the absence of disease or infirmity” [4]. Wellbeing is a dynamic construct comprised of several dimensions. In a cohomprensive view of wellness can be defined 3 main domain of wellness: the psycological health, the physical health and the subjective wellbeing. Subjective wellbeing (SWB) is often measured via validated psychometric scales, and individual and community surveys. Subjective wellbeing is linked to Health-Related Quality of Life (HRQoL) but is not synonymous with it. The factors identified as the most important for subjective wellbeing vary across space, time and cultural context (Fig. 2).

Wellness entails contemporary also the simultaneous fulfillment of the three types of needs. Personal needs (e.g., health, self-determination, meaning, spirituality, and opportunities for growth), are intimately tied to the satisfaction of collective needs such as adequate health care, environmental protection, welfare policies, and a measure of economic equality; for citizens require public resources to pursue private aspirations and maintain their health. Wellness also concerns relational needs. Two sets of needs are primordial in pursuing healthy relationships among individuals and groups: respect for diversity and collaboration and democratic participation.

Most approaches to community wellbeing (or its associated terms) follow a components approach: the majority of them have, at their core, an emphasis on individual wellbeing. PULSE has focused on defining and developing a new concept of Urban Wellbeing tied to the broader concept of Urban Health Resilience. This recognizes the connections between the physical characteristics of the urban environment (including assets and deficits) and human health (including both physical and psychological health). The PULSE concept of Urban Wellbeing refers to the interaction between the positive and negative experiences within cities (whether objective or subjective), and the individual and community practices of mobility and placemaking. This novel interpretation of Wellbeing focuses on the dynamic interplay between individual psychological characteristics and strengths, neighborhoods in which people live and work, and the capacity of individuals to respond to environmental and interpersonal stressors [6]. Within our population urban health model, the physical and social environments are understood as key drivers of Wellbeing. This prioritizes an integrated, or relational, approach to urban places and health equity, including population differences in Wellbeing. Central to this relational approach is the idea that place matters – that our health and wellbeing are shaped by the characteristics of the settings where we live and work, and these environments are in turn shaped by our health-related actions and behaviours. Several recent studies have highlighted this important dynamic. Using data from the English Longitudinal Study of Aging, Hamer and Shankar [7] found that individuals who hold more negative perceptions of their neighbourhood report less positive Wellbeing, and experience a greater decline in Wellbeing over time.

Of course, place itself can have a profound impact on our Wellbeing.

### Urban Resilience and Wellbeing

In PULSE, we contextualize wellbeing within a model of urban resilience:

Urban resilience refers to the ability of an urban system - and all its constituent socio-ecological and socio-technical networks across temporal and spatial scales – to maintain or rapidly return to desired functions in the face of a disturbance, to adapt to change, and to quickly transform systems that limit current or future adaptive capacity. In this definition, urban resilience is dynamic and offers multiple pathways to resilience (e.g., persistence, transition, and transformation). It recognizes the importance of temporal scale, and advocates general adaptability rather than specific adaptedness. The urban system is conceptualized as complex and adaptive, and it is composed of socio-ecological and socio-technical networks that extend across multiple spatial scales. Resilience is framed as an explicitly desirable state and, therefore, should be negotiated among those who enact it empirically.

Resilient urban neighborhoods can be broadly defined as those that have lower than expected premature mortality (measured via the Urban Health Indicators).

In PULSE, we define Urban Wellbeing as an integral component of Urban Resilience. Urban Wellbeing, in this context, refers to the individual traits and capacities to prepare for, respond to, and recover from the personal and interpersonal challenges encountered in cities. These challenges could include experiences of bias and exclusion, on the one hand, and exposure to under-resourced or polluted environments, on the other. Each of these challenges is associated with physiological and psychological stress at the individual and community level. Stress is, of course, antithetical to Wellbeing. Translating this concepts into data constructs two main instruments are available into PULSE architecture: the risk assessment models, previously described and the urban maps.

### Urban Maps

The physical environment, socio-economic and cultural conditions, urban planning, available public or private services and leisure facilities are some of the factors that can have an effect on a person’s health. Hence, an interest in the study of geographical patterns of health-related phenomena has increased in recent years. Within this context, maps have been demonstrated to be a useful tool for showing the spatial distribution of many types of data used in public health in a visual and concise manner [13, 14]. For example, it permits the study of general geographical patterns in health data and identifying specific high-risk locations. An example of these maps in PULSE are the personal exposure maps. Personal exposure is a concept from the epidemiological science to quantify the amount of pollution that each individual is exposed to, as a consequence of the living environment, habits etc.

Personal exposure has been obtained matching the data from the dense network of low-cost sensors and the informations on habits coming from the PulsAIR app. Following the sampling rate of the sensors the data has been calculated.

**Fig. 3.**
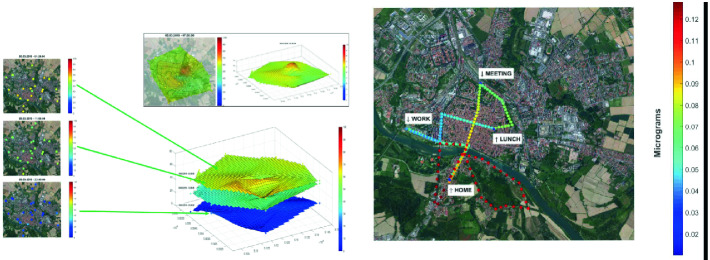
Personal exposure map to PM10

Figure 3 shows a map for the personal exposure to PM10 with an hourly frequency.

Furthermore using the GPS tracks from the PulsAIR app, FitBit and the personal exposure, an estimate of inhaled pollutant has been obtain in association to three classes of movement by the speed of body translation; standing, walking and running, considering the breaths per minute and the air volume per breath [15].

Personal exposure result has been also traced into exposure paths as in Fig. 3: a time-lapse of 1 min correspond to a dot movement line.

## Conclusions

The multivariate data driven approach of PULSE gives an example of a new conception of health and wellness, not only focused on individual health status, but also on the relationship between individual and environment. Such vision can be also directed toward the definition of “planetary health” provided by “The Lancet Contdown” [16]. The data driven approach pursuited in PULSE has surely given a great opportunity to implement such a vision, that maybe would not so immediatiate without possibility to integrate different sources of data.
